# Supplementary material for: MassWateR: Improving quality control, analysis, and sharing of water quality data
Source: PLoS One. 2023 Nov 1;18(11):e0293737. doi: 10.1371/journal.pone.0293737 (PMC10619867; doi:10.1371/journal.pone.0293737)
Supplement: S1 File — (DOCX) [file pone.0293737.s001.docx]

*Supplement* MassWateR: Improving quality control, analysis, and sharing of water quality data

Marcus W Beck^1,*^, Benjamen Wetherill^2^, and Jillian Carr^3^

^1^ Tampa Bay Estuary Program, St. Petersburg, Florida, USA
^2^ ACASAK Consulting, Boston, Massachusetts USA
^3^ Massachusetts Bays National Estuary Partnership, Boston, Massachusetts, USA

^*^ Correspondence: [Marcus W Beck <mbeck@tbep.org>](mailto:mbeck@tbep.org)

Table S1: Complete list of parameters and units that can be used with MassWateR. Parameters can be named using the Simple Parameter or the WQX Parameter convention. Only one unit of measure can be used per parameter. WQX: Water Quality Exchange.

| **Simple Parameter** | **WQX Parameter** | **Units of measure** |
| --- | --- | --- |
| Air Temp | Temperature, air | deg C, deg F |
| Ammonia | Ammonia | mg/l, ug/l, umol/l, ppm |
| Ammonium | Ammonium | mg/l, ug/l, umol/l, ppm |
| Chl a | Chlorophyll a | mg/l, ug/l, umol/l, ppm |
| Chl a (probe) | Chlorophyll a (probe) | mg/l, ug/l, umol/l, ppm |
| Chloride | Chloride | mg/l, ug/l, umol/l, ppm |
| Conductivity | Conductivity | uS/cm, mS/cm, S/m |
| Cyanobacteria (lab) | Algae, blue-green (phylum cyanophyta) density | mg/l, ug/l, umol/l, ppm |
| Cyanobacteria (probe) | Chlorophyll a (probe) concentration, Cyanobacteria (bluegreen) | mg/l, ug/l, umol/l, ppm |
| Depth | Depth | m, cm, ft |
| DO | Dissolved oxygen (DO) | mg/l, ug/l |
| DO saturation | Dissolved oxygen saturation | % |
| E.coli | Escherichia coli | cfu/100ml, MPN/100ml, #/100ml |
| Enterococcus | Enterococcus | cfu/100ml, MPN/100ml, #/100ml |
| Fecal Coliform | Fecal Coliform | cfu/100ml, MPN/100ml, #/100ml |
| Flow | Flow | cfs, cfm, mgd, l/sec, l/min, |
| Gage | Height, gage | m, cm, ft |
| Metals | Metals | mg/l, ug/l, umol/l, ppm |
| Microcystins | Microcystins | mg/l, ug/l, umol/l, ppm |
| Nitrate | Nitrate | mg/l, ug/l, umol/l, ppm |
| Nitrate + Nitrite | Nitrate + Nitrite | mg/l, ug/l, umol/l, ppm |
| Nitrite | Nitrite | mg/l, ug/l, umol/l, ppm |
| Ortho P | Orthophosphate | mg/l, ug/l, umol/l, ppm |
| pH | pH | blank, s.u., None |
| Pheophytin | Pheophytin a | mg/l, ug/l, umol/l, ppm |
| POC | Particulate organic carbon | mg/l, ug/l, umol/l, ppm |
| PON | Total Nitrogen, mixed forms | mg/l, ug/l, umol/l, ppm |
| POP | Phosphorus, Particulate Organic | mg/l, ug/l, umol/l, ppm |
| Salinity | Salinity | ppth, PSU, PSS, g/kg, ppt |
| Secchi Depth | Depth, Secchi disk depth | m, cm, ft |
| Silicate | Silicate | mg/l, ug/l, umol/l, ppm |
| Sp Conductance | Specific conductance | uS/cm, mS/cm, S/m |
| Sulfate | Sulfate | mg/l, ug/l, umol/l, ppm |
| Surfactants | Surfactants | mg/l, ug/l, umol/l, ppm |
| TDN | Total Nitrogen, mixed forms | mg/l, ug/l, umol/l, ppm |
| TDP | Total Phosphorus, mixed forms | mg/l, ug/l, umol/l, ppm |
| TDS | Total dissolved solids | mg/l, ug/l, umol/l, ppm |
| TKN | Total Kjeldahl nitrogen | mg/l, ug/l, umol/l, ppm |
| TN | Total Nitrogen, mixed forms | mg/l, ug/l, umol/l, ppm |
| TP | Total Phosphorus, mixed forms | mg/l, ug/l, umol/l, ppm |
| TSS | Total suspended solids | mg/l, ug/l, umol/l, ppm |
| Turbidity | Turbidity | FTU, FNU, JTU, NTU, AU, BU, FAU, FBU, FNMU, FNRU, NTMU, NTRU |
| Water Temp | Temperature, water | deg C, deg F |

Table S2: The sample file included with MassWateR for the data quality objectives for accuracy.

| **Parameter** | **uom** | **MDL** | **UQL** | **Value Range** | **Field Duplicate** | **Lab Duplicate** | **Field Blank** | **Lab Blank** | **Spike/Check Accuracy** |
| --- | --- | --- | --- | --- | --- | --- | --- | --- | --- |
| Water Temp | deg C | NA | NA | all | <= 1.0 | <= 1.0 | NA | NA | <= 1.0 |
| pH | NA | NA | NA | all | <= 0.5 | <= 0.5 | NA | NA | <= 0.2 |
| DO | mg/l | NA | NA | < 4 | < 20% | NA | NA | NA | NA |
| DO | mg/l | NA | NA | >= 4 | < 10% | NA | NA | NA | NA |
| Sp Conductance | uS/cm | NA | NA | < 250 | < 30% | < 30% | NA | <= 50 | <= 50 |
| Sp Conductance | uS/cm | NA | 10000 | >= 250 | < 20% | < 20% | NA | <= 50 | <= 50 |
| TP | mg/l | 0.01 | NA | < 0.05 | <= 0.01 | <= 0.01 | BDL | BDL | <= 0.01 |
| TP | mg/l | 0.01 | NA | >= 0.05 | < 30% | < 20% | BDL | BDL | <= 15% |
| Nitrate | mg/l | 0.05 | NA | all | < 30% | < 20% | BDL | BDL | <= 15% |
| Ammonia | mg/l | 0.10 | NA | all | < 30% | < 20% | BDL | BDL | <= 15% |
| E.coli | MPN/100ml | 1.00 | NA | <50 | < log30% | < log30% | BDL | BDL | NA |
| E.coli | MPN/100ml | 1.00 | NA | >=50 | < log20% | < log20% | BDL | BDL | NA |

Table S3: The sample file included with MassWateR for the data quality objectives for frequency and completeness.

| **Parameter** | **Field Duplicate** | **Lab Duplicate** | **Field Blank** | **Lab Blank** | **Spike/Check Accuracy** | **% Completeness** |
| --- | --- | --- | --- | --- | --- | --- |
| Water Temp | 10 | 10 | NA | NA | 10 | 90 |
| pH | 10 | 10 | NA | NA | 10 | 90 |
| DO | 10 | NA | NA | NA | NA | 90 |
| Sp Conductance | 10 | 10 | NA | 10 | 10 | 90 |
| TP | 10 | 5 | 10 | 5 | 5 | 90 |
| Nitrate | 10 | 5 | 10 | 5 | 5 | 90 |
| Ammonia | 10 | 5 | 10 | 5 | 5 | 90 |
| E.coli | 10 | 5 | 10 | 5 | NA | 90 |
