# Supplementary material for: MassWateR: Improving quality control, analysis, and sharing of water quality data
Source: PLoS One. 2023 Nov 1;18(11):e0293737. doi: 10.1371/journal.pone.0293737 (PMC10619867; doi:10.1371/journal.pone.0293737)
Supplement: Attachment — Submitted filename: review-response.docx [file pone.0293737.s002.docx]

**Response**: We sincerely thank the academic editor and two reviewers for providing thoughtful comments on our manuscript. We provide a point-by-point response to these comments below. We hope that these responses and changes to the manuscript are satisfactory.

## Academic Editor

Though one of the reviewers recommended a complete rejection, I found that this study could be of benefit to the community especially in the industry 4.0 era. However, I request that you take into consideration the following while revising your manuscript.

1. You should describe explicitly how the package provides a better alternative to all the other existing packages. Where applicable, cite the weaknesses of the existing packages with supporting citations.

**Response**: Thank you for this comment. As noted in the original submission on line 88, there are no other R packages that we are aware of that provide similar functionality. As such, there is no basis of comparison for MassWateR to determine relative strengths/weakness with other packages. However, we have added text describing other R packages that are somewhat related and have included a description of software packages outside of the R environment to provide a more comprehensive discussion of alternatives. The following additions were made:

“The *TADA* R package (Mullin et al. 2022) is also currently under development as a resource for compiling and evaluating data from the WQP. *TADA* is similar to *dataRetrieval* in that it can be used for importing data, but the package is also expected to provide more comprehensive methods for cleaning, filtering, and processing data using the rich qualifier codes provided by WQP.”

“Other software platforms outside of the R environment provide various services for quality control of water quality data. For example, the [Aquarius](https://aquaticinformatics.com/products/aquarius-environmental-water-data-management/) software environment is a proprietary resource for managing hydrologic and water quality data and is used by several private and public institutions. Functionality is provided to pre-process and synthesize multiple data streams, manually correct erroneous values, and to visualize results for decision-making. A graphical user interface is provided to access the various features of the software, as compared to a programmatic approach for building custom routines. The software is not open-source, which implicitly limits its development to a core set of maintainers and is not freely accessible to the broader community (e.g., Schrape 2017). Similar platforms are available from companies that manufacture data loggers (e.g., [YSI](https://www.ysi.com/), [HOBO](https://www.onsetcomp.com/products/software/hoboware)), all of which are specific to the monitoring equipment and not broadly transferable. Alternative publicly funded software and data services are provided by the Consortium of Universities for the Advancement of Hydrologic Science ([CUAHSI](https://www.cuahsi.org/data-services/solutions)). Services provided by CUAHSI include data discovery, archiving, cloud computing, and analysis, with many of the services available in open source environments (e.g., [Python](https://www.python.org/)). However, the services provided by CUAHSI are meant to address a variety of different resources and use cases, none of which are immediately related to quality control reporting and data submission to public institutions. As such, specific software solutions to address these needs and that leverage existing tools available in a rich open-source environment are clearly needed.”

2. Make appropriate citations to literature sources. For example, in L26 and L29, you talk about an Act and an EU framework whose sources are not cited. The same applies to L39-40, where you argue out why this communication could be important but without any supporting citation(s). Similar omissions are evident in the DISCUSSION where you should make appropriate citation of the “community of practice established” and any outcomes (L521-537, and so forth).

**Response**: We have added citations where needed.

Line 26 includes a link to a description of the US Clean Water Act legislation ([33 USC § 1251 et seq.](https://uscode.house.gov/view.xhtml?path=/prelim@title33/chapter26&edition=prelim), note that there is no citation provided in the references because this is legislative code).

Line 29 includes a citation to the European Water Framework Directive (Directive 2000/60/EC of the European Parliament and of the Council of 23 October 2000, Pollard and Huxham 1998)

Line 40 includes the following citations: Kinzelman et al. (2003), Wagner et al. (2006), Zhang (2006).

Lines 521-537 include the following citations: Meyer et al. (2022), Whitehead (2007), List, Ebert, and Albrecht (2017), Wickham (2016)

Additional citations were added later in the discussion: DeCicco et al. (2022), Wagner et al. (2006)

3. L87-99 appears to should have been part of the introduction, highlighting what the MassWateR brings onboard.

**Response**: This paragraph and the additions shown above were moved to the introduction.

4. Figure 1 could best be presented as a flow/block diagram.

**Response**: Figure 1 has been reconfigured as a flow diagram, although emphasis remains in the text that a user does not have to engage in all steps of the workflow.

5. For Table 4, it would be important to provide all the parameters tested as a supplementary file to support the submission. Please ensure that the full details of the algorithms designed are provided, and where there are restrictions, it should otherwise be stated.

**Response**: A supplement file was created to accompany the main manuscript, including tables for the parameters used by MassWateR and the sample files included with the package for the data quality objectives for accuracy, frequency, and completeness. Also note that the specific QC checks used by the package were previously described in the main text (e.g., lab spikes, lab blanks, etc.).

6. To improve the chances of manuscript suitability for PLOS ONE, ensure that the criteria listed for manuscript types (https://journals.plos.org/plosone/s/submission-guidelines#loc-methods-software-databases-and-tools) are met. i.e. Utility, Validation and Availability for articles describing new/improved METHODS, SOFTWARE, DATABASES, and TOOLS.

**Response**: We have verified that our manuscript adheres to the utility, validation, and availability criteria for the article type. Below are descriptions of how these criteria are met:

*Utility:* The MassWateR package provides needed utility for the community, as established in the introductory text, both in the original submission and the additions provided in the revision. In particular, the package provides a repeatable and efficient means for generating QC reports to ensure water quality data are of sufficient accuracy and precision needed for integration into larger databases (i.e., WQX) or additional applications (e.g., regulatory, assessment). As noted, we are unaware of any existing tools that provide this functionality.

*Validation*: MassWateR achieves its intended uses as verified by adoption by the larger community of practice, detailed in the text. In addition, the package has been downloaded over 4000 times since its availability on CRAN in January 2023. This provides a robust indication that those outside of our community of practice are also applying MassWateR for its intended use. Finally, we describe the possible applications for the package in detail in the manuscript (e.g., QC report generation including a figure showing a portion of the report, submission to WQX, etc.).

*Availability*: The software is entirely open-source and available for download through [CRAN](https://cran.r-project.org/package=MassWateR), [GitHub](https://github.com/massbays-tech/MassWateR), and [R-Universe](https://fawda123.r-universe.dev/MassWateR) Numerous links and citations are included throughout the text. The license applied to MassWateR is CC0 1.0, making the code available to the world-wide public domain.

## Reviewer 1

The paper describes a new R package for the processing of water quality information, mainly following US standards, but could be adapted for other regions. It is well written and easy to follow. I have only a few minor comments. Several of the figures were missing from the submission, so I could not check them (Figure 3, and Figure 5 following).

**Response**: Thank you for your review comments. We have provided responses below. Please note that some of the figures provided in the journal submission system can only be viewed by selecting the download link on the page for each figure. I have verified that all figures were present in the original submission and the revision.

L27 and 29 please cite respective regulatory frameworks

**Response**: References were added for both the Clean Water Act and Water Framework Directive.

L108 should be “their data in several ways”

**Response**: Accepted suggestion.

L109 should be “outlier checks”

**Response**: Edited the sentence as follows: “The first step, QC screening, is often iterative as a user can modify parts of the raw data based on messages from the data import functions or checks for outliers.”

L137 should be function - spelling error

**Response**: Corrected.

L327 should be “to evaluate”

**Response**: Edited the sentence as follows: “The QC checks for frequency and completeness can be used to evaluate if…”

L334-335 I guess more correct would be to state “to verify that the parameter is below a certain threshold, e.g. below detection limit”. Even if the parameter has a value of zero - the parameter itself is not absent

**Response**: Edited as follows: “Measurements in the field to verify that the parameter is below a certain threshold, e.g., below the detection limit of the field equipment”

336-337 same

**Response**: Edited as follows: “Laboratory samples to verify that the parameter is below a certain threshold, e.g., below the detection limit of the laboratory analysis method”

338-339 More correct would be “to assess the similarity of values, i.e. precision is high. (Precision is a value that is calculated from bot measurements - do not understand how each measurement can have its own precision)

**Response**: Accepted suggestion and made a similar edit for lab duplicates.

385 Add: “in red in Table 3 show…”

**Response**: Accepted suggestion.

439 spelling “can be”

**Response**: Corrected.

506 I guess public use is better than public consumption

**Response**: Edited the sentence as follows: “…and made accessible for public use.”

## Reviewer 2

The availability of reliable monitoring data is the basis of scientific research. This study collected discrete surface water quality data generated by MassWateR software package and analyzed the trend of parameters. The study has some fundamental significance. However, this paper is more like a technical manual than a scientific research paper. Therefore, I would recommend the authors to submit the paper to a technical journal rather than a research-oriented journal.

**Response**: Thank you for taking the time to review our paper. Based on the opinion of the academic editor, this paper is considered to be of sufficient interest to the readership of the journal. Additionally, the paper is not written as a research article and was submitted as a software manuscript under the acceptable formats considered by the journal (see [here](https://journals.plos.org/plosone/s/submission-guidelines#loc-methods-software-databases-and-tools)). The format of these submissions is notably different from those of conventional research articles. In particular, papers describing software must demonstrate utility, validation, and availability. Please see our comment to the academic editor above regarding these requirements.

In addition, the transferability of the developed tool needs to be demonstrated and analyzed, which is directly related to its application value. The authors should give concrete examples in this area;

**Response**: Thank you for this suggestion. In our original submission, we provided substantial text describing how MassWateR can be used in more generic applications, i.e., its transferability. This information was provided in the discussion on lines 538-556, where specific considerations were noted for how users could apply the software to novel datasets. Truthfully, we are not yet aware of specific applications of MassWateR outside of our core user base, but our intention of publishing this work in the primary literature was to reach a broader audience and thereby promote transferability of the software. We are confident that the software can be broadly applied given the relatively minor limitations for its use outside of Massachusetts. Additionally, the software has been downloaded over 4000 times from CRAN as of this writing, which suggests many others have found the software transferable.

Finally, the quality of the authors’ images is so poor that improving the clarity is very necessary.

**Response**: Please note that the article submission system greatly reduces the quality of the submitted figures at the end of the manuscript. The original, high-resolution figures can be viewed by clicking the link on the top of the page for each figure. Several are also provided as .svg files, following the journal guidelines. Previews for these figures are not provided in the PDF and they can only be viewed by downloading each file.

# References

DeCicco, Laura A., David Lorenz, Robert M. Hirsch, William Watkins, and Mike Johnson. 2022. *dataRetrieval: R packages for discovering and retrieving water data available from U.S. federal hydrologic web services* (version 2.7.12). Reston, VA: U.S. Geological Survey; U.S. Geological Survey. <https://doi.org/10.5066/P9X4L3GE>.

Kinzelman, Julie, Clement Ng, Emma Jackson, Stephen Gradus, and Robert Bagley. 2003. “Enterococci as Indicators of Lake Michigan Recreational Water Quality: Comparison of Two Methodologies and Their Impacts on Public Health Regulatory Events.” *Applied and Environmental Microbiology* 69 (1): 92–96. <https://doi.org/10.1128/aem.69.1.92-96.2003>.

List, Markus, Peter Ebert, and Felipe Albrecht. 2017. “Ten Simple Rules for Developing Usable Software in Computational Biology.” Edited by Scott Markel. *PLOS Computational Biology* 13 (1): e1005265. <https://doi.org/10.1371/journal.pcbi.1005265>.

Meyer, Michael F, Carolina C Barbosa, Robert Ladwig, Jorrit P Mesman, Nahit Soner Börekçi, Kaelin Cawley, Johannes Feldbauer, et al. 2022. “Hacking Limnology Workshop and DSOS22: Creating a Community of Practice for the Nexus of Data Science, Open Science, and the Aquatic Sciences.” *Limnology & Oceanography Bulletin* 31 (4): 123–26. <https://doi.org/10.1002/lob.10525>.

Mullin, Cristina A., Jacob Greif, Michelle Thawley, Laura Shumway, Justin Bousquin, Laura Decicco, Katie Healy, Zach Smith, Florian Rupprecht, and Brad Cooper. 2022. *Water Quality Portal (WQP) Tools for Automated Data Analysis (TADA)*. Washington, DC: U.S. Environmental Protection Agency. <https://github.com/USEPA/TADA>.

Pollard, Peter, and Mark Huxham. 1998. “The European Water Framework Directive: A New Era in the Management of Aquatic Ecosystem Health?” *Aquatic Conservation: Marine and Freshwater Ecosystems* 8 (6): 773–92. <https://doi.org/10.1002/(sici)1099-0755(1998110)8:6<773::aid-aqc313>3.0.co;2-r>.

Schrape, Jan-Felix. 2017. “Open-Source Projects as Incubators of Innovation: From Niche Phenomenon to Integral Part of the Industry.” *Convergence: The International Journal of Research into New Media Technologies* 25 (3): 409–27. <https://doi.org/10.1177/1354856517735795>.

Wagner, Richard J, Robert W Boulger Jr, Carolyn J Oblinger, and Brett A Smith. 2006. “Guidelines and Standard Procedures for Continuous Water-Quality Monitors: Station Operation, Record Computation, and Data Reporting.” Techniques and Methods 1-D3. Version 1.0. Reston, VA: U.S. Geological Survey. <https://doi.org/10.3133/tm1D3>.

Whitehead, Jim. 2007. “Collaboration in Software Engineering: A Roadmap.” *Future of Software Engineering (FOSE ’07)*, May. <https://doi.org/10.1109/fose.2007.4>.

Wickham, Hadley. 2016. *Ggplot2: Elegant Graphics for Data Analysis*. Springer-Verlag New York. <https://ggplot2.tidyverse.org>.

Zhang, C. 2006. *Fundamentals of Environmental Sampling and Analysis*. Wiley. <https://doi.org/10.1002/0470120681>.
