# Supplementary material for: MassWateR: Improving quality control, analysis, and sharing of water quality data
Source: PLoS One. 2023 Nov 1;18(11):e0293737. doi: 10.1371/journal.pone.0293737 (PMC10619867; doi:10.1371/journal.pone.0293737)
Supplement: Attachment — Submitted filename: review-response.docx [file pone.0293737.s003.docx]

**Response**: We again thank the reviewers for evaluating our manuscript. Please see our responses below. Note that reviewer 1 suggested publication without further comment.

## Reviewer 2

The authors have improved over the previous version, but there are still some issues that need to be revised, the main comments are as follows:

1. It is recommended to add information about the data of the mean values as well as information about the true values in Figures 3 and 6, so that the reader can understand more information;

- **Response**: Figure 3 shows boxplot distributions of selected parameters to aid package users in identifying potential outlier observations. Boxplots are common visualization tools that can quickly demonstrate central tendency and spread of a group of observations (Tukey et al. 1977; McGill, Tukey, and Larsen 1978), in addition to outliers as intended by the plot in Figure 3. Therefore, we do not consider it necessary to show mean values in this plot. Further, Figure 6 currently shows mean values. The values shown are considered “true” as these are sampled values from *in situ* or laboratory-based methods and not those from modeled or other predictive techniques.

2. The quality of Figure 4 is very low and it is not even possible to see any content information;

- **Response**: As noted in our responses to comments in the previous revision, the figure quality is substantially reduced in the PDF that is generated by the article submission system. High-resolution figures were included with the submission and can be downloaded by selecting the link that appears at the top-right of each page for each figure.

3. The dimensions of Figure 8 are recommended to use vertical arrangement; and the scale labeling form is not standardized; the DO color labeling colors of the sites are more distinguishable;

- **Response**: The figures produced by *MassWateR* are all ggplot class objects and can be modified using conventional *ggplot2* functions. A vertical arrangement can be achieved as follows, assuming the reviewer means that the legend should appear above and arranged horizontally on the plot.
- library(ggplot2)

  anlzMWRmap(fset = fsetls, param = "DO", addwater = "medium") +
   theme(legend.position = 'top')

- The online website includes a vignette about modifying *MassWateR* plots with *ggplot2* code. We have added the following text on line 445 to make this clear.
- “Additionally, all plots returned by *MassWateR* are ggplot class objects and can be modified accordingly using conventional *ggplot2* functions (see the website [vignette](https://massbays-tech.github.io/MassWateR/articles/modifying.html)).”

4. How do the predictions of water quality parameters in this study compare with other common models?

- **Response**: The functions in *MassWateR* rely on data collected *in situ* and there are no aspects related to predictive modeling included in the package. The primary goals of *MassWateR* are to improve how environmental professionals perform quality control, analysis, and sharing of monitoring data. As such, the intent is to support the use of data for environmental decision-making. No predictions are provided by the package.

5. Some recent deep learning models for water quality prediction should be mentioned, e.g., 10.1016/j.ejrh.2023.101331; 10.1007/s11783-023-1688-y; 10.1016/j.ecolind.2023.109882; 10.1016/j.jhydrol.2023. 129649.

- **Response**: Please see our response to the previous comment.

# References

McGill, Robert, John W. Tukey, and Wayne A. Larsen. 1978. “Variations of Box Plots.” *The American Statistician* 32 (1): 12. <https://doi.org/10.2307/2683468>.

Tukey, John W et al. 1977. *Exploratory Data Analysis*. Vol. 2. Reading, MA.
